# Short sleep duration and obesity among Australian children

**DOI:** 10.1186/1471-2458-10-609

**Published:** 2010-10-15

**Authors:** Zumin Shi, Anne W Taylor, Tiffany K Gill, Jane Tuckerman, Robert Adams, James Martin

**Affiliations:** 1 Population Research and Outcome Studies Unit, Department of Health, South Australia; 2 Department of Medicine, University of Adelaide, South Australia; 3 Department of Pulmonary Medicine, Women's and Children's Hospital, South Australia

## Abstract

**Background:**

There is limited information on sleep duration and obesity among Australian children. The objective of the study is to cross-sectionally examine the relationship between sleep duration and obesity in Australian children aged 5 to 15 years.

**Methods:**

Data were collected using the South Australian Monitoring and Surveillance System between January 2004 and December 2008. Each month a representative random sample of South Australians are selected from the Electronic White Pages with interviews conducted using Computer Assisted Telephone Interviewing (CATI). Within each household, the person who was last to have a birthday was selected for interview. Parents reported the number of hours their children slept each day. Obesity was defined according to the International Obesity Task Force (IOTF) definition based on BMI calculated from reported body weight and height.

**Results:**

Overall, parents of 3495 children aged 5-15 years (mean 10.7 years, 50.3% boys) were interviewed. The prevalence of obesity was 7.7% (8.9% in boys, 6.6% in girls). In multivariate analysis after adjusting for sociodemographic variables, intake of fruit and vegetables, physical activity and inactivity, the odds ratio (OR) for obesity comparing sleeping <9 hours with ≥10 hours was 2.23 (95% CI 1.04-4.76) among boys, 1.70(0.78-3.73) among girls, and 1.97(1.15-3.38) in both genders. The association between short sleep (<9 hours) and obesity was stronger in the younger age group. No significant association between short sleep and obesity was found among children aged 13-15. There was also an additive interaction between short sleep and low level of physical activity.

**Conclusion:**

Short sleep duration is associated with increased obesity in children especially among younger age groups and boys.

## Background

Sleep, like physical activity and diet, serves an important role in the growth, maturation, and health of the child and adolescent [1]. Sleep deprivation is becoming prevalent in both adults and children. A short sleep duration is related to decreased levels of leptin, glucose tolerance, insulin sensitivity, but increased levels of ghrelin, hunger and appetite [2-7]. It is also associated with behaviors that are known to promote weight gain and obesity including lower physical activity and lower fruit and vegetable consumption [8]. Cumulative evidence shows that short sleep duration is related to the risk of obesity [9,10]. Targeting short sleep duration may offer a novel and effective method of preventing and treating obesity [11].

In recent years, an increasing number of population studies on sleep and obesity among children have become available. However, the age ranges within the published studies are usually small [12-19], with few studies reporting an age range >5 years [20-22]. A recent systematic review shows that children aged <10 years demonstrate an inverse association between sleep duration and overweight/obesity [23]. A 32-year prospective birth cohort study shows short sleep during childhood (5-11 years of age) was associated with an increased risk of adult obesity, but sleep time at 32 years of age was not associated with adult BMI [22]. It is not clear whether 10 years of age is the critical cut off in the association between sleep duration and obesity. However, such information is important in order to identify high risk groups for intervention.

There is limited information on sleep duration and obesity among Australian children. One study reported an association between short sleep and increased risk of overweight among Australian boys but not girls. However, the study was based on data collected in 1985, and there was no adjustment for fruit and vegetable consumption and socio-economic variables [20]. In another study, declines in sleep duration were found for both girls (28 min) and boys (33 min) aged 10-15 years in Australia from 1985 to 2004 [24]. The prevalence of obesity among children in Australia also increased in the same period. Updated research is needed.

Using data from the South Australian Monitoring and Surveillance System (SAMSS), the objective of this study was to assess the association between sleep duration and obesity among children aged 5-15 years. The second objective of the study was to examine the interaction between sleep and activity and the effect on obesity. We hypothesized that 1) there is an association between short sleep duration and obesity; 2) there is an interaction between sleep and physical activity/inactivity in relation to obesity.

## Methods

### Survey design and sample selection

Data for this study were collected using the South Australian Monitoring and Surveillance System (SAMSS) from January 2004 to December 2008. SAMSS is designed to systematically monitor the trends of diseases, health related problems, risk factors and other health services issues for all ages over time for the South Australian (SA) health system [25]. Interviews are conducted on a minimum of 600 randomly selected people (of all ages) each month. All households in SA with a telephone connected and the telephone number listed in the Electronic White Pages (EWP) are eligible for selection in the sample. A letter introducing the survey is sent to the selected household and the person with the last birthday is chosen for interview. There are no replacements for non-respondents. Up to ten call backs are made to the household to interview the selected persons. Interviews are conducted by trained health interviewers. SAMSS utilises a Computer Assisted Telephone Interviewing (CATI) system to conduct the interviews. Data are weighted by area (metropolitan/rural), age, gender and probability of selection in the household to the most recent SA population data so that the results are representative of the SA population [26]. For participants aged less than 16 years, data are collected from an adult in the household who has been nominated by a household member as the most appropriate adult to answer questions on the child's behalf.

In the period January 2004 to December 2008 a total of 3505 interviews were conducted (the overall response rate was 68.2%) with parents of children aged 5-15 years old. Children with a sleep duration <4 or >14 hours/day were excluded from the analysis (n = 10). A total of 3,495 children were included in the analysis. Ethical approval for the project was obtained from University of Adelaide (ethics approval number H-182-2009). All participants gave informed consent.

### Data items

*Sleep duration *was assessed using the question "How many hours per day does your child spend sleeping?" Sleep duration was categorized as follows: <9 hours, 9-10 hours, and ≥10 hours.

*Body mass index *(BMI) was derived from parents-reported weight and height. Obesity was defined according to the International Obesity Task Force (IOTF) definition [27].

*Fruit and vegetable consumption *was assessed by asking parents how many serves of fruit and vegetables their children usually ate per day.

*Physical activity and inactivity *were assessed by a set of questions. "On average, how many hours per day or per week does the child spend doing organised sport, reading for pleasure, studying/doing homework at home, watching TV, videos or playing video or computer games?"

#### Demographic variables

Sex, age, area of residence and gross annual household income were included in the analyses.

### Data analyses

Chi square tests were used to compare differences in categorical variables. The association between sleep duration and the risk of obesity was analysed using logistic regression models, adjusting for multiple covariates. The logistic model controlled for age (continuous), gender, income, residence, physical activity/inactivity and intake of fruit and vegetables. Multiplicative interaction between sleep duration and age/sex was tested using the Wald test by adding the product of sleep and age/sex in the multivariate model. Statistical significance was considered if p < 0.05 (two sided). All analyses were undertaken using STATA survey commands (SVY) (Version 10, StataCorp, College Station, TX).

## Results

**Figure 1:**
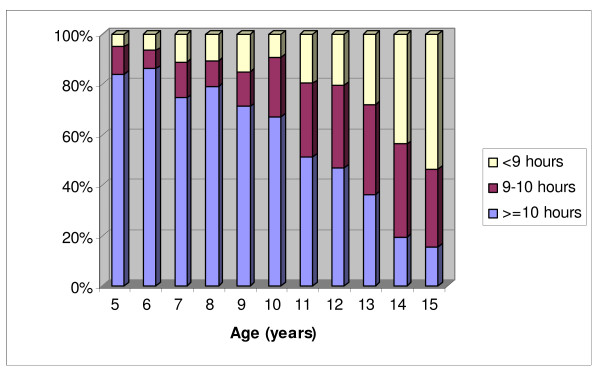
Distribution of sleep duration among children aged 5-15 years in South Australia

Among 3495 children aged 5-15 years (mean 10.7 years, 50.3% boys), the prevalence of obesity was 7.7% (8.9% in boys, 6.6% in girls). The mean duration of sleep was 9.5 hours. In total, 23.9% of the participants had sleep duration of less than 9 hours, while 51.8% reported sleeping 10 hours or more everyday. Sleep duration was inversely associated with age (p < 0.001) (Figure 1).

The prevalence of obesity was significantly different among sleep duration groups (Table 1). A strong relationship between a short sleep duration (<9 hours) and obesity was found among children aged 5-10 years (younger age group). Among the younger age group, comparing sleeping less than 9 hours with 10 hours or more, the prevalence of obesity almost doubled (22.3% versus 11.5%). Among children aged 13-15 years, no such association was found. Short sleep duration was significantly associated with physical inactivity, physical activity, and intake of fruit but not vegetables.

**Table 1:** Sample characteristic across sleep duration groups among children aged 5-15 years in South Australia (SA), January 2004 to December 2008

	≥10 hours (n = 1812)	9-10 hours (n = 848)	<9 hours (n = 835)	P
Age (SD)	9.2(2.9)	12.0(2.7)	12.7(2.6)	<0.001
Boys (%)	50.7	51.5	48.1	0.621
Obesity (%)	8.9	5.2	7.8	0.004
Age 5-10 years (n = 1574)	11.5	12.9	22.3	0.032
Age 11-12 years (n = 631)	3.3	3.6	10.3	0.067
Age 13-15 years (n = 1290)	4.1	2.2	3.2	0.606

BMI (mean, SE)	18.0(0.14)	19.5(0.20)	20.4(0.27)	<0.001
Household income (%)				
>20000$	87.0	86.3	82.5	
up to 20000$	5.6	7.2	8.6	0.013
not stated	7.3	6.5	8.9	

Area of residence (%)				
Metro Adelaide	67.6	70.4	75.7	<0.001
SA country	32.4	29.6	24.3	

Intake of vegetable (servings/day) (mean, SE)	2.2(0.04)	2.4(0.07)	2.3(0.07)	0.113
Intake of fruit (servings/day) (mean, SE)	1.8(0.03)	1.7(0.06)	1.6(0.05)	0.001
Hours on sport per day(mean, SE)	0.4(0.02)	0.6(0.03)	0.7(0.06)	<0.001
Hours on reading for pleasure per day(mean, SE)	0.5(0.02)	0.6(0.03)	0.7(0.05)	0.001
Hours on studying/home work at home per day(mean, SE)	0.5(0.03)	0.7(0.04)	0.9(0.04)	<0.001
Hours on TV, video, computer games(mean, SE)	1.7(0.04)	1.9(0.06)	2.1(0.08)	<0.001

SE = standard error, BMI = body mass index

P-value is from comparison across groups of sleep duration using ANOVA (continuous variables), or chi-square test (categorical variables)

Using multivariate analysis (Table 2), after adjusting for socio-demographic factors, physical activity and intake of fruit and vegetable, short sleep duration was significantly associated with obesity in boys but not girls. The odds ratios (OR) for obesity comparing sleeping <9 hours with ≥10 hours were 2.23 (95% CI: 1.04-4.76) among boys, 1.70 (0.78-3.73) among girls, and 1.97 (1.15-3.38) for both genders.

**Table 2:** Odds ratio (OR) and 95% confidence interval (CI) for obesity according to sleep duration in children aged 5-15 living in South Australia

	≥10 hours (n = 1812)	9-10 hours (n = 848)	<9 hours (n = 835)
**Gender**			
Boys			
Age adjusted	1	1.11(0.54-2.27)	2.23(1.08-4.62)
Multivariate adjusted ^a^	1	1.15(0.55-2.39)	2.23(1.04-4.76)
Girls			
Age adjusted	1	1.03(0.48-2.20)	1.81(0.85-3.85)
Multivariate adjusted ^a^	1	0.97(0.44-2.14)	1.70(0.78-3.73)
Both genders ^b^			
Age adjusted	1	1.07(0.63-1.81)	2.03(1.20-3.44)
Multivariate adjusted ^a^	1	1.04(0.61-1.78)	1.97(1.15-3.38)
**Age groups**			
5-12 years			
Age, gender adjusted	1	1.18(0.68-2.06)	2.61(1.52-4.50)
Multivariate adjusted ^a^	1	1.18(0.67-2.09)	2.52(1.44-4.41)
13-15 years			
Age, gender adjusted	1	0.46(0.13-1.66)	0.56(0.17-1.88)
Multivariate adjusted ^a^	1	0.47(0.14-1.50)	0.47(0.15-1.50)

^a ^Adjusted for age, physical activity and inactivity, household income, intake of fruit and vegetable, area of residence (metro Adelaide, SA country)

^b ^Also adjusted for gender

p = 0.367 for interaction between age and sleep. P = 0.369 for interaction between gender and sleep.

Stratified analysis showed the association between short sleep and obesity was stronger in the younger age group. There were no significant multiplicative interactions between sleep duration and age (p = 0.367) or sleep duration and gender (p = 0.369). As a large proportion of children aged 13 and above had sleep duration <9 hours, we undertook additional analysis comparing <8 hours of sleep with ≥10 hours of sleep, and found no significant association between sleep duration and the risk of obesity among this age group (data not shown).

**Figure 2:**
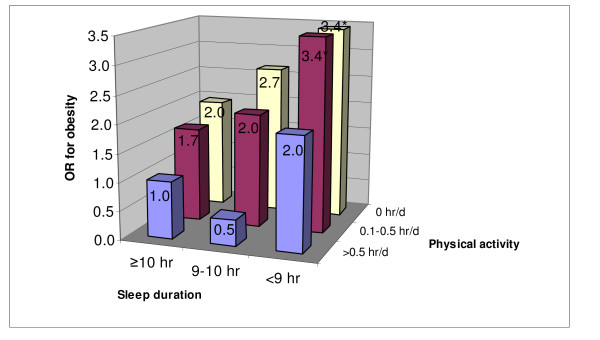
**Joint effects of sleep duration and physical activity on the risk of obesity among children aged 5-15 years in South Australia**. OR adjusted for age, gender, physical inactivity, household income, intake of fruit and vegetables, area of residence (metro Adelaide or SA country). * p < 0.05

Physical activity was inversely associated with obesity. In multivariate analysis, using hours of organized physical activity as a continuous variable, the OR for obesity was 0.57 (0.33-1.00). As gender was not significant in the multivariate model, all respondents were combined in a multivariate analysis and the joint effect of sleep and organized physical activity was examined. Based on a visual inspection of the odds ratios in Figure 2, there appears to be an additive interaction between sleep duration and physical activity was found. Children with both short sleep and low level of physical activity had the highest risk of obesity (Figure 2). Compared with those ≥0.5 hours of daily physical activity and sleep duration >10 hours, children with <9 hours of sleep and daily physical activity <0.5 hour had 3.4 times (95%CI 1.31-9.02, p = 0.012) higher the risk of obesity.

## Discussion

In this cross-sectional study we found that a short sleep duration was associated with an increased risk of obesity among children aged 5-15 years living in South Australia. Although a trend was seen for a stronger effect in boys than girls, no significant difference was found between the two groups. Low levels of physical activity added an additional risk of obesity to short sleep duration. Adjusting for socioeconomic status, lifestyle factors, and fruit and vegetable consumption did not change this association.

Several mechanisms may be involved in this association, including decreased glucose tolerance, decreased insulin sensitivity, elevated sympathovagal balance, increased evening concentrations of cortisol, increased levels of ghrelin, decreased levels of leptin, and increased hunger and appetite [2-6]. In human studies, short sleep is related to increased fat intake [28] and increased intake of energy from snacks [29]. Short sleep duration is also associated with risk behaviors that are known to promote weight gain and obesity including lower physical activity and lower fruit and vegetable consumption [8].

The findings of the study are consistent with previous studies on sleep and obesity among children as well as adults as shown in recent reviews [10,23]. The association between sleep and obesity was stronger among younger age group (below 12 years), however the interaction between age and sleep was not significant. This is slightly different from the findings in the review by Chen et al [23], which shows 10 years of age is the limit for an association between sleep and increased risk of obesity. However in a previous study in Australian children [20] the OR for obesity and a short sleep duration was much higher among boys aged 14.0 to 16.5 years than boys aged (7.5 to 13.9 years). The reason for this difference is unclear. However, based on findings from most of the studies, interventions targeting adequate sleep to prevent obesity should focus on younger age groups.

Although most studies reported no gender difference in the association between sleep duration and obesity [12,14,16,18,22,30], some studies in the USA [21] and Australia [20] observed such difference. In our study, stratified analysis showed that the OR for obesity in those with short sleep was greater than 1 among girls, but it was not statistically significant. However, in the multivariate model gender was not significantly associated with obesity and the multiplication interaction between gender and sleep was not significant. It has been suggested that girls may be more resilient to environmental stressors and need greater sleep deprivation in order to be affected compared to boys [20,31]. Further research is needed to clarify whether the gender difference exists.

Most studies use physical activity as a confounding factor when assessing the association between sleep duration and obesity [19,21,32], although it might be more appropriate to be treated as an effect modifier. No study has specifically looked at the interaction between sleep and physical activity in relation to obesity. We found an additive interaction between short sleep duration and physical activity. Children with low levels of physical activity and short sleep had 3.4 times higher the OR of obesity. These results emphasize the importance of both sleep and physical activity in the prevention of childhood obesity.

There are a number of potential shortcomings of this study. The cross-sectional study design precludes the establishment of causal relationships. We are not able to adjust for puberty status, which is shown to be related to the risk of obesity [33]. Furthermore, the dietary pattern was not measured. In a population study it has been shown that short sleep is related to higher intake fat [28]. Although it is commonly used, the validity of parental reporting of sleep duration and BMI is unknown. The quality of sleep was not measured. Another limitation is that no difference in sleep duration between week days and weekend days was examined. Other studies have shown that people catch up their sleep debt during weekends [34]. The strength of this study is that it is based on a representative population sample with a wide age range.

## Conclusion

In summary, the current study confirms that short sleep duration is associated with obesity among children in Australia, especially boys aged 5-12 years. Low levels of physical activity added an additional risk to short sleep duration. Regardless of the cause and effect relationship, given the existing evidence, encouraging adequate sleep among children is important in the prevention of obesity.

## Abbreviations

SAMSS: South Australian Monitoring and Surveillance System; OR: odds ratio

## Competing interests

The authors declare that they have no competing interests.

## Authors' contributions

AWT, TKG and ZS participated in the concept and design of the study. ZS, AWT, JT, RA, JM participated in the interpretation of data and revision of the paper. ZS analysed the data and wrote the report. The corresponding author had full access to all data in the study and had final responsibility for the decision to submit for publication. All authors read and approved the final manuscript.

## Pre-publication history

The pre-publication history for this paper can be accessed here:

http://www.biomedcentral.com/1471-2458/10/609/prepub
